# Importance of Pharmacogenetics and Drug–Drug Interactions in a Kidney Transplanted Patient

**DOI:** 10.3390/life13081627

**Published:** 2023-07-26

**Authors:** Julia Concha, Estela Sangüesa, Ana M. Saez-Benito, Ignacio Aznar, Nuria Berenguer, Loreto Saez-Benito, M. Pilar Ribate, Cristina B. García

**Affiliations:** Facultad de Ciencias de la Salud, Universidad San Jorge, Villanueva de Gállego, E-50830 Zaragoza, Spain; jconcha@usj.es (J.C.); esanguesa@usj.es (E.S.); amsaezbenito@usj.es (A.M.S.-B.); i.aznar@pssjd.org (I.A.); nberenguer@usj.es (N.B.); lsaezbenito@usj.es (L.S.-B.); cbgarcia@usj.es (C.B.G.)

**Keywords:** tacrolimus, omeprazole, renal transplant, pharmacogenetics, polymorphisms, drug–drug interaction

## Abstract

Tacrolimus (TAC) is a narrow-therapeutic-range immunosuppressant drug used after organ transplantation. A therapeutic failure is possible if drug levels are not within the therapeutic range after the first year of treatment. Pharmacogenetic variants and drug–drug interactions (DDIs) are involved. We describe a patient case of a young man (16 years old) with a renal transplant receiving therapy including TAC, mycophenolic acid (MFA), prednisone and omeprazole for prophylaxis of gastric and duodenal ulceration. The patient showed great fluctuation in TAC blood concentration/oral dose ratio, as well as pharmacotherapy adverse effects (AEs) and frequent diarrhea episodes. Additionally, decreased kidney function was found. A pharmacotherapeutic follow-up, including pharmacogenetic analysis, was carried out. The selection of the genes studied was based on the previous literature (*CYP3A5*, *CYP3A4*, *POR*, *ABCB1*, *PXR* and *CYP2C19*). A drug interaction with omeprazole was reported and the nephrologist switched to rabeprazole. A lower TAC concentration/dose ratio was achieved, and the patient’s condition improved. In addition, the TTT haplotype of ATP Binding Cassette Subfamily B member 1 (*ABCB1*) and Pregnane X Receptor (*PXR*) gene variants seemed to affect TAC pharmacotherapy in the studied patient and could explain the occurrence of long-term adverse effects post-transplantation. These findings suggest that polymorphic variants and co-treatments must be considered in order to achieve the effectiveness of the immunosuppressive therapy with TAC, especially when polymedicated patients are involved. Moreover, pharmacogenetics could influence the drug concentration at the cellular level, both in lymphocyte and in renal tissue, and should be explored in future studies.

## 1. Introduction

Tacrolimus (TAC) is among the most used immunosuppressive drugs after an allogenic solid organ transplantation [1]. This therapy is usually combined with glucocorticoids, mycophenolic acid (MFA), azathioprine or mTOR inhibitors [2]. TAC is a macrolide that prevents the transcription of a specific group of lymphokine genes, finally avoiding the formation of cytotoxic lymphocytes, which are mainly responsible for organ rejection. This calcineurin inhibitor has a narrow therapeutic range, being able to produce failure of the therapy or conversely adverse effects (AEs). As the interindividual variability of drug exposure is wide and multifactorial, it is essential to maintain TAC blood levels within the therapeutic range and to measure it regularly after transplantation. For this reason, drug levels—trough concentration, Co 12 h post administration—must be monitored over time, usually at least every 6 months in long-term transplantation. This drug extensively binds to erythrocytes, so hematocrit affects the whole-blood trough concentrations. Moreover, 99% of TAC is bound to albumin and x1-acidclycoprotein in plasma, whereas <1% of it is freely distributed [3]. Although unbound TAC is the pharmacologically active part of the drug, the measurement of this free fraction involves technical difficulties [4]. Recently, new methods have been developed for the measurement of free levels in plasma, but at the moment there is not any standardized target plasma concentration, and their implications must be investigated before being carried out in clinical practice [5].

TAC is absorbed all over the gastrointestinal tract, mainly in the small intestine, controlled by p-glycoprotein (p-gp), also called Multidrug Resistance Protein 1 (MDR1), which is an efflux pump located in the apical membrane of enterocytes that regulates the passage of some xenobiotics across cellular membranes. P-gp is also expressed in hepatocytes and renal tubular epithelial cells, determining biliary and renal elimination [1]. A recent review highlighted the importance of this transporter for TAC levels within the lymphocyte, the pharmacological target [1]. TAC is widely metabolized by the hepatic cytochromes CYP3A5 and CYP3A4, also present in the intestine [6]. Foods can affect the bioavailability of this drug, especially those rich in fat. TAC AEs are documented in the literature as very being frequent, including hyperglycemia, muscle pain, nephrotoxicity and gastrointestinal effects (diarrhea) [7].

The use of pharmacogenetics as a predictor of the response to immunosuppressive therapy has been shown to be clinically relevant. Several studies have pointed out the significant difference in the bioavailability of TAC in the presence of certain single-nucleotide polymorphisms (SNPs), as they influence the expression and function of relevant proteins, and the influence of many genes has been previously analyzed [8,9,10,11,12]. The use of concomitant medications has also been shown to affect immunosuppressive therapy as it is a source of potential drug–drug interactions (DDIs), and it is very common that transplanted patients are polymedicated. Specifically, DDIs between the proton pump inhibitor (PPI) omeprazole and CYP3A4 or p-gp substrates have been reported [13]. Omeprazole is mainly metabolized by the CYP2C19 and CYP3A4 cytochromes. Likewise, it is a p-gp substrate and inhibitor [14,15]. In this sense, omeprazole is a candidate to interact with TAC by the CYP3A4 or p-gp route, as previous cases have described [16,17,18,19].

Here, we describe the case of a DDI in a polymedicated renal transplant patient in which pharmacogenetic mechanisms were probably also involved. The patient was suffering AEs after renal transplantation and the possible causes were explored. The objective of this case study is to evaluate the effect of polymorphisms and DDI on the pharmacotherapy of TAC in a renal transplant patient.

## 2. Materials and Methods

### 2.1. Ethical Approval and Informed Consent

The development of this study was approved by the regional Ethical Committee, Research Ethics Committee of the Autonomous Community of Aragon (CEICA). Informed consent was obtained and signed by the legal tutors.

The patient’s tolerance and adherence to treatment was examined and confirmed through interviews.

### 2.2. Case Presentation

A 16-year-old European, white male received a renal transplantation with an initial immunosuppressant pharmacotherapy consisting of immediate-release TAC (0.5 mg-0-1 mg) corresponding to 0.02 mg/kg, 360 mg MFA (1-0-1), 5 mg prednisone (1-0-0) and 20 mg omeprazole per day (1-0-0). The patient presented significant episodes of muscle pain and diarrhea after transplantation, some of them resulting in hospitalization. Prior to transplantation, his gastrointestinal function was regular. Diarrhea is defined as an increase in frequency and a decrease in stool consistency compared to usual. The glomerular filtration rate (GFR) was low. The patient had no family history of transplant or other concomitant disease. The optimal TAC blood level for this patient, according to the nephrologist, should be 4–7 ng/mL, since a Symphony study predefined this range as the target concentration, resulting in better allograft function and reduced AEs while maintaining efficacy [20,21].

On suspicion of a DDI with omeprazole, the nephrologist switched to rabeprazole.

### 2.3. TAC Pharmacokinetics

TAC blood levels were monitored every 6 months by microparticle chemiluminescent immunoassay (MEIA, Abbott Diagnostics, Chicago, IL, USA). The assay detection limit was 0.3 ng/mL at the 95% confidence level as performed on the IMx analytical platform, including before and after switching to rabeprazole. Concomitant drugs (MFA, omeprazole, prednisone) were not measured during clinical practice at the patient’s hospital.

Creatinine was measured by enzymatic assay. GFR was estimated from creatinine levels.

### 2.4. DNA Isolation and Genotyping

A literature review of genetic variables related to TAC therapy was performed and *CYP3A5*, *CYP3A4* and *ABCB1* resulted as the main variants, and they were analyzed. TAC is mainly metabolized by CYP3A5 and CYP3A4 and transported by p-gp [22,23,24,25,26,27]. *CYP2C19* was also analyzed according to the Zhao et al. (2012) procedure [15] because of the possible omeprazole interaction (as its main metabolizing pathway). In addition, other polymorphisms explored in previous studies that are related to TAC pharmacotherapy were included [28].

Genomic DNA was extracted from peripheral whole-blood samples using Whatman FTA™ cards and incubation with Chelex-100 resin (Bio-Rad^®^ Laboratories, Hercules, CA, USA).

A total of 12 SNPs of 6 genes were analyzed: c.6986A > G of the *CYP3A5* gene (NM_000777.5); g.-290A > G, c.15389C > T and g.87925_87926insA (p.Pro488Thr*fs494) of the CYP3A4 gene (NM_001202855.3); c.3435T > C (p.Ile1145=), c.2677T>G/A (p.Ala893Thr/Ser) and c.1236T > C (p.Gly412=) of the *ABCB1* gene (NM_000927.3); c.63396C > T and c.69789A > G of Pregnane X Receptor (*PXR*) (NM_022002.2); and c.1508C > T (p.Ala503Val) of the cytochrome P450 oxidoreductase (*POR*) gene (NM_000941.3) related to TAC metabolism and transport. The C.681G > A (p.Pro227=) and c.636G > A (p.Trp212*) variants of the *CYP2C19* gene (NM_000769.4) related to omeprazole metabolism were also analyzed.

Genotyping was performed using Polymerase Chain Reaction Restriction Fragment Length Polymorphism (PCR-RFLP; MJ MiniTM Personal Thermal Cycler Bio-Rad^®^ Laboratories, Hercules, CA, USA) or sequencing methods. Specific TaqMan™ (Applied Biosystems, Foster City, CA, USA) or rhAmp^®^ (IDT, Newark, NJ, USA) genotyping systems were carried out on CFX Connect™Systems (Bio-Rad Laboratories, Hercules, CA, USA) for the validation of results. Specific techniques and conditions for each SNP are detailed in Table 1.

Statistical analysis (correlation) was performed using SPSS^®^ (Statistical Package for the Social Sciences) software version 25 (IBM^®^, Armonk, NY, USA).

## 3. Results

### 3.1. TAC Pharmacokinetics

Despite continuous dosage readjustments (TACₜₒₜ oral dose ranging from 2 to 3.5 mg/day), TAC blood levels suffered larger fluctuations that did not follow a linear relation regarding the oral dose, and a high concentration/dose ratio was produced.

**Figure 1:**
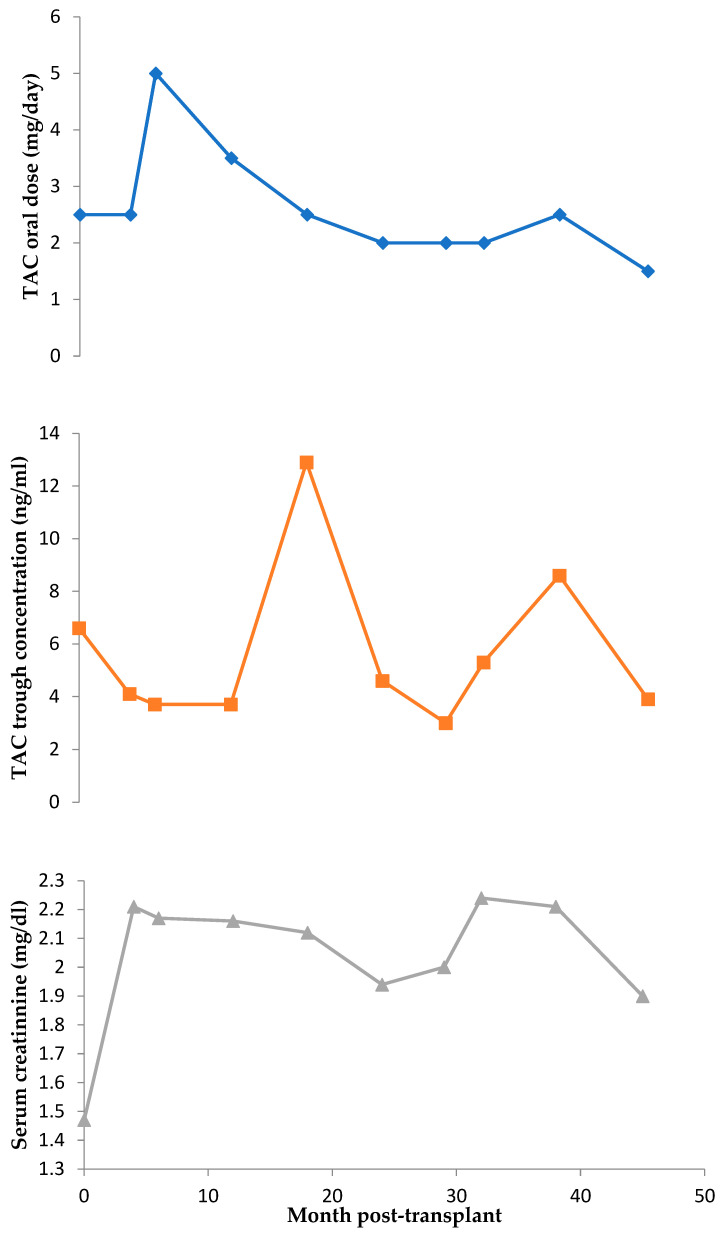
Timeline of tacrolimus (TAC) trough concentration (ng/mL), serum creatinine levels (mg/dL) and TAC oral dose (mg/day).

When TAC therapy started, creatinine serum levels increased directly proportional to the TAC dose, producing a chronic moderate stage 3 renal failure (minimum estimated GFR 32.8 mL/min/1.73 m^2^ when taking 2 mg TAC/day). Figure 1 shows the evolution over time of TAC trough concentrations and creatinine serum levels according to the TAC oral dose.

**Figure 2:**
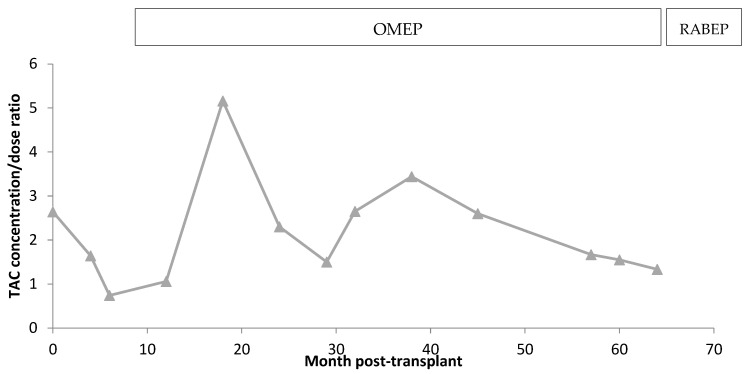
Comparation of TAC concentration/dose ratio with omeprazole and rabeprazole. Omep = omeprazole; Rabep = rabeprazole.

After the PPI change, the blood levels of TAC followed a linear relation with respect to the oral dose (R^2^ = 0.9944) and had a lower average value. Moreover, the change from omeprazole to rabeprazole was beneficial for the patient, since AEs decreased, pointing to a possible DDI. Figure 2 shows the evolution of TAC concentration/dose ratio with both PPIs.

### 3.2. Genotype

The obtained genotypes for the patient and their associated phenotypic characteristics are shown in Table 2. Since there are some mutations in the patient, pharmacogenetics could be important to explain the occurrence of AEs.

## 4. Discussion

### 4.1. Clinical Variants Affecting Short-Term Transplantation Pharmacokinetics

The pharmacokinetics of tacrolimus may change a long time after transplantation and are affected by many factors, with special variations in short-term post-transplantation [37]. In addition to genotype, co-treatments, patient age, time post-transplantation, hematocrit, albumin, and liver and kidney functions are influencing factors [38]. Hematocrit tends to change in the period after kidney transplantation, being low and increasing substantially after the first months post-transplantation as kidney recovery occurs and erythropoietin levels increase [39]. It is not clear how TAC pharmacokinetics during this post-transplant period are explained by alterations in hematocrit [40]. As TAC is highly bound to erythrocytes, hematocrit has been shown to affect TAC whole-blood concentrations, while free-plasma TAC (active) is not altered. A low hematocrit increases TAC whole-blood clearance [37,39].

This could potentially lead to an incorrect dose adjustment and the appearance of inconsistent correlations between the whole-blood concentration and AEs [40,41]. Moreover, young patients have a decreased drug binding affinity for plasma proteins, with an increased free drug fraction with respect to the whole-blood TAC [42]. In our patient, these variables could have been the cause of such decreased TAC trough levels despite a low oral dose in the first months post-transplantation. However, the presence of AEs corresponding to therapeutic trough level TAC concentrations during long-term post-transplantation needs further explanation.

### 4.2. Patient’s Pharmacogenetics and Drug–Drug Interactions

Several genetic variants have been described to influence TAC therapy, highlighting those involved in its metabolism and transport. The most relevant variant affecting TAC metabolism is c.6986A > G of the *CYP3A5* gene, which results in a premature stop, generating a non-functional enzyme [7,8]. However, this variant is highly variable between ethnicities, being predominantly present in 94% of the European population, but in just 18% of Africans and 67% of South Asians [9].

The Clinical Pharmacogenetics Implementation Consortium guidelines (CPIC) have established that *CYP3A5* expressers (c.6986AA) need a 1.5~2-fold dose of TAC compared to non-expressers (c.6986GG) (level 1A of evidence) [43]. In this last group, CYP3A4 is mainly responsible for TAC metabolism. The *CYP3A4* gene has two main variants: g.-290A > G increases the enzyme expression by altering the binding affinity of various transcription factors, while c.15389C > T generates reduced mRNA production, altering the enzyme activity [10,11]. The recently discovered rare “Spanish variant”, g.87925^g.87926insA, generates an adenosine insertion and a premature stop codon (p.Pro488Thr*494) [31].

**Figure 3:**
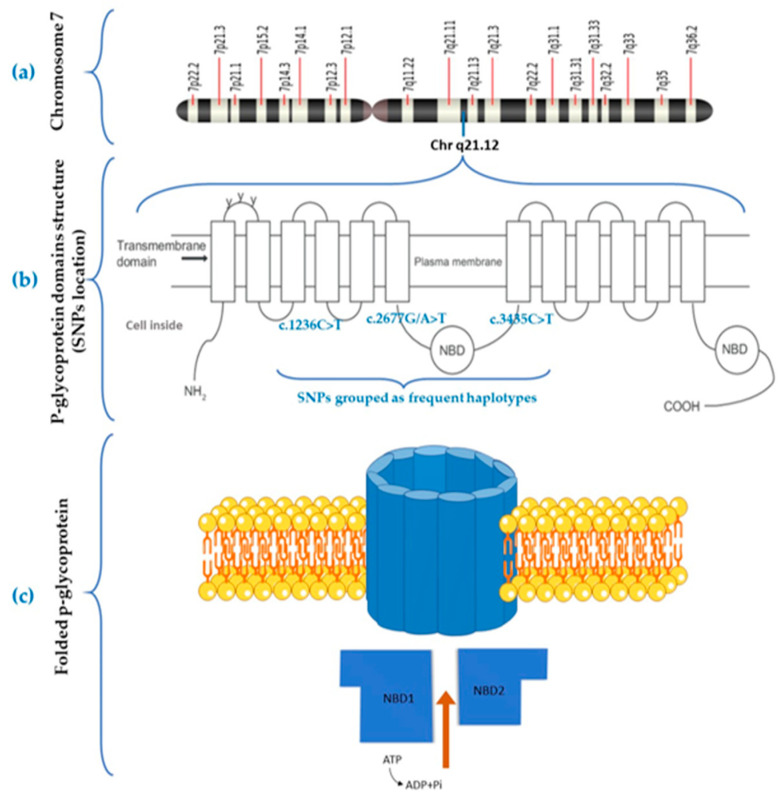
Chromosome 7, *ABCB1* haplotype location, and p-gp structure. (**a**) Chromosome 7 structure and *ABCB1* gene location; (**b**) c.1236C > T, c.2677G/A > T and c.3435C > T single-nucleotide polymorphisms (SNPs) location in unfolded p-gp; (**c**) folded p-gp structure and two nucleotide-binding domains (NBDs). Source: Adapted figure from (**a**) National Library of Medicine (NLM), (**b**) Adapted with permission from Ref. [46] Copyright 2023, Dove Press. (**c**) own source.

Moreover, the P450 oxidoreductase, encoded by the *POR* gene and necessary for CYP oxidation, presents the c.1508C > T variant, which generates rapid metabolizers in CYP3A-expressers, having been previously associated with lower TAC blood levels [5,32,44]. The *ABCB1* gene, which encodes for p-gp, presents three main variants: two silent polymorphisms (c.3435C > T and c.1236C > T) and a polymorphism in the promoter region (c.2677G/A > T). These three variants are often grouped as haplotypes, as they are in a strong linkage disequilibrium and tend to be inherited together [45]. The location of these variants and the structure of p-gp is shown in Figure 3.

Previous studies have highlighted the superiority of haplotype analysis versus individual variants to predict the *ABCB1* gene phenotype [47]. Most of the observed haplotypes in most ethnicities for these three SNPs are CGC containing Ala in the 893 position and TTT containing Ser in the 893 position [33]. Related to the above, the presence of the TTT haplotype has been demonstrated to decrease p-gp activity, allowing a greater entry of oral drugs across the gastrointestinal tract as well as other cellular membranes expressing this protein [5,48,49,50].

Moreover, the *Pregnane X Receptor (PXR)* gene, also the *NR1I2* gene, encodes for Pregnane X Receptor, which is a transcription factor implicated in *CYP3A* and *ABCB1* regulation [5,27]. The c.69789A > G variant of *PXR* generates lower *PXR* mRNA expression and therefore decreased p-gp and CYP3A4 activity, while the c.63396C > T variant produces enhanced *PXR* mRNA expression [34,35].

Related to omeprazole, the *CYP2C19* gene, encoding for the CYP2C19 enzyme, presents two main variants that generate loss-of-function alleles. C.681G > A generates a splicing defect (p.Pro227=), while c.636G > A produces a truncated protein (p.Trp212*) [36]. The metabolism of rabeprazole is less dependent on oxidation via CYP2C19 than other PPIs; as a result, there are minimal differences in clearance and exposure in patients who are fast or slow metabolizers of this cytochrome [51]. The patient presented the wildtype genotype for the *CYP3A4* and *POR* genes, as well as the homozygous CYP3A5*3 genotype, so these do not seem to play a role in the occurrence of AEs. Furthermore, the patient showed no genetic variants decreasing CYP2C19 metabolism. Similar cases have been reported involving pharmacological interactions between TAC and omeprazole. These were produced by the displacement of the metabolism of omeprazole to CYP3A4, secondary to CYP2C19 malfunction, producing a competitive interaction [15,16,17,18]. Zhao et al. (2012) [15] described a similar case report in a 17-year-old renal transplant patient. According to their scheme of action, a possible interaction with omeprazole was suspected to explain the TAC blood level fluctuations.

Accordingly, another study showed that *CYP2C19*-mutated patients co-administered with TAC and omeprazole presented a higher concentration/dose ratio compared to the wildtype [52]. Omeprazole was replaced by rabeprazole in accordance with the nephrologist, which does not report interactions with TAC according to the literature. However, a pharmacogenetic analysis revealed the wildtype *CYP2C19* genotype. Additionally, other cases reported a TAC–omeprazole interaction in *CYP2C19* wildtype patients, with CYP3A4 and p-gp inhibition being the cause of the DDI [16,17,18]. Specifically, the one carried out by Maguire et al. (2012) considered rabeprazole as a better option in kidney transplant recipients with respect to other PPIs due to intestinal p-gp inhibition and the increased oral bioavailability of TAC [17]. Nevertheless, other studies have concluded that the TAC–omeprazole interaction is not clinically relevant in renal transplant patients [53,54]. Recently, an extensive review of predictors of TAC pharmacokinetic variability showed that PPIs (omeprazole, lansoprazole, esomeprazole) can increase TAC concentration by up to 2- to 3-fold, recommending rabeprazole as a safer alternative [55]. In our study, the change from omeprazole to rabeprazole was beneficial for the patient, but *CYP2C19* mutation was not the cause.

The patient presented the TTT haplotype in homozygosis for the *ABCB1* gene in addition to the *PXR* c.69789A > G variant. Both produced the reduced expression and activity of p-gp, decreasing its effectiveness as an efflux pump and allowing a greater passage of TAC through cell membranes, including the intestine and kidney. Further, and although there are few reports on the matter, the involvement of this protein in TAC levels within the lymphocyte has been studied, since it seems to be directly related to the therapeutic efficacy of the drug rather than blood levels. The presence of the TTT haplotype seems to produce a greater therapeutic effect and a lower oral dose needed compared to the wildtype, as increased levels have been observed [50,56]. Recent reviews have focused on the importance of *ABCB1* pharmacogenetic biomarkers and transplant therapy outcome, although the relationship between intracellular concentrations and whole-blood levels needs further investigation [57,58,59]. Specifically, the Degraeve et al. (2020) [55] review highlighted the importance of *ABCB1* SNPs on local cellular concentration (lymphocyte and kidney) and therapy outcomes. *PXR* expression could also affect TAC levels in lymphocytes, since these cells have been shown to express this gene [60]. The malfunction of this protein can increase the bioavailability of TAC in whole blood and lymphocytes, as well as the potential to generate AEs, especially nephrotoxicity, due to renal tissue accumulation. Previous clinical studies have indicated that p-gp inhibitors affect TAC bioavailability more than its clearance, showing the key role of this membrane protein [16].

**Figure 4:**
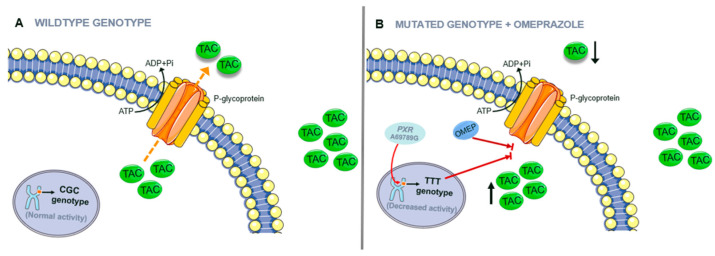
Influence of genotype on drug pharmacokinetics: (**A**) Wildtype genotype producing regular tacrolimus (TAC) active ejection; (**B**) TTT haplotype and Pregnane X Receptor (PXR) 69789A > G plus the presence of omeprazole inhibit TAC ejection. Omeprazole (OMEP); adenosine triphosphate (ATP); adenosine diphosphate (ADP). Source: own elaboration.

Figure 4 shows how the TTT haplotype and *PXR* 69789A > G variants plus omeprazole p-gp inhibition affect p-gp efficiency.

Nevertheless, rabeprazole does not produce a p-gp inhibition like omeprazole, allowing this efflux pump to eject a greater amount of the drug [14]. The switching to rabeprazole produced a lower concentration/dose ratio and the expected lower TAC levels in lymphocytes compared with omeprazole therapy in our patient [61]. Diarrhea and muscle pain disappeared, and decreased creatinine levels were also observed. There exist few studies analyzing TAC concentrations inside the kidney. Between the two found, only one demonstrated that higher renal TAC levels analyzed by biopsy were related to nephrotoxicity. However, they found no associations with *CYP3A5* nor the *ABCB1* genotype [62]. Other studies in mice showed a relationship between *ABCB1* genetics and the tissue–blood concentration ratio, but in the liver [63].

One limitation of our study is the lack of physiological data that may affect TAC blood levels, such as hematocrit, alpha-1 acid glycoprotein or albumin. More limitations are the scarce data regarding the patient’s evolution of the TAC concentration/dose ratio and creatinine levels after the change to rabeprazole, or the lack of monitoring of MYC concentrations as another possible cause of the diarrhea episodes. Kidney function after transplantation is frequently suboptimal and is not an AE caused by the pharmacotherapy.

The lack of knowledge and resources, or the irrelevance that is sometimes given to pharmacogenetics and DDI can lead to therapeutic inefficiencies, either in the case of immunosuppressive drugs such as TAC or in other fields. Specifically, PPIs are often prescribed together with immunosuppressive therapy due to the high incidence of gastric and duodenal ulcerations that can occur with drugs such as mycophenolic acid or corticosteroids (up to 39%) [17]. Most current attending physicians have not obtained training in pharmacogenetics or there are simply no established protocols for it. However, the new generations of practitioners seem to be more trained in the area and show interest in its direct application to the patient [64]. Despite this, these tools are little established in the daily clinical practice of most hospitals, or in the case of specific and scarce drugs.

## 5. Conclusions

We can conclude that considering the individual genetics and co-treatment of transplanted patients is a focus point to avoiding AEs during immunosuppressant pharmacotherapy. Ulcers are serious complications that can be prevented using PPIs, which have a low economic cost. Omeprazole is the most prescribed PPI for the prophylaxis of gastric and duodenal ulceration and can be the cause of unexpected pharmacological DDIs by pharmacogenetic pathways. Although its prescription is not strictly justified in the presence of corticosteroids, this is the reason why they are often prescribed in conjunction with immunosuppressive therapy.

In our patient, switching to rabeprazole allowed the improvement of gastrointestinal function and muscle pain as well as the decrease in creatinine levels. A higher TAC concentration/dose ratio was observed for omeprazole with respect to rabeprazole therapy, showing the potential interaction of omeprazole with p-gp.

Despite that this association cannot be demonstrated with a single case report, previous studies have been able to demonstrate it, although there is still some controversy in this regard. We suggest rabeprazole may be a safer option in these cases. The *ABCB1* gene is not commonly being studied in clinical practice, although it plays an important role in TAC pharmacotherapy, as has been shown for other drugs (i.e., chemotherapeutic agents). Influential genes such as *PXR* can also affect the activity of key proteins such as p-gp or P450-cytochromes, although more evidence is still needed. However, only variant c.6986A > G of the *CYP3A5* gene has a 1A level of evidence and is present in the CPIC guidelines for TAC. In this case report, the inhibition of p-gp by the *ABCB1* TTT haplotype plus *PXR* c.69789A > G variants and omeprazole could explain the existence of long-term TAC AEs during post-transplantation, while the TAC trough levels were within the range expected. Intracellular levels seemed to be affected by p-gp. The presence of all these factors probably produces a summative or even synergistic effect on the activity and expression of p-gp. The influence of omeprazole could be observed from the decrease in TAC concentration/dose ratio when switching to rabeprazole. 

Definitely, one of the most promising fields still to be explored in renal transplant pharmacotherapy with TAC is the influence of drug concentration at the cellular level, both in lymphocyte and in renal tissue. Pharmacogenetics aims to be a key point in this field. More research is needed to assess this relationship within a large sample size of patients.

In this case report, pharmacogenetics did not drive the decision to change the drug since the pharmacological interaction was already present, but rather allowed the causes of this interaction to be determined and a safe pharmacological alternative to be chosen. The genetic profile of an individual is unchanged, so it should be part of the patient’s clinical history and contribute to future decision making and clinical practice based on pharmacogenetics. Two genes, which are not generally analyzed, were found to be relevant in this case report.

We propose that these variants should be considered in clinical practice, especially in drugs with a narrow therapeutic margin, such as TAC, although more clinical evidence is needed. Pharmacogenetics may have a great impact on the efficacy of treatment with this type of drug, and it is especially important in vulnerable individuals such as transplanted patients. Certainly, this would be a fundamental first step to start applying the possibilities offered by pharmacogenetics in the individualized optimization of the patient’s pharmacotherapy.

## Figures and Tables

**Table 1:** Methods, primers, and enzyme (if applicable) of the analyzed polymorphisms.

Gene	Variant	Analysis Technique	Primers (Sequencing or PCR-RFLP)	Enzyme	References
CYP3A5 (NM_000777.5)	c.6986A > G	Sequencing and rhAmp	F: 5′-ACTGCCCTTGCAGCATTTAG-3′ R: 5′-CCAGGAAGCCAGACTTTGAT-3′		[22]
CYP3A4 (NM_001202855.3)	g.-290A > G	Sequencing and rhAmp	F: 5′-CAGAAGGGATGACATGCAGA-3′ F: 5′-GGAAGAGGCTTCTCCACCTT-3′		[23]
*CYP3A4* (NM_001202855.3)	c.15389G > T	TaqMan	ID Assay C__59013445_10		
*CYP3A4* (NM_001202855.3)	c.1461_1462 insA	Sequencing	F: 5′-GAAGGAGTGTCTCACTCA-3′ R: 5′-GAGGTCTCTGGTGTTCTCAG-3′		[24]
*POR* (NM_000941.3)	c.1508C > T	Sequencing and rhAmp	F: 5′-CATCTGTGCGGTGGTTGT-3′ R: 5′-TGAAGGGCAGGCGGA-3′		
*ABCB1* (NM_000927.3)	c.3435C > T	PCR-RFLP and rhAmp	F: 5′-GATCTGTGAACTCTTGTTTT-3′ R: 5′-GAAGAGAGACTTACATTAGGC-3′	*MboI*	[25]
*ABCB1* (NM_000927.3)	c.1236C > T	PCR-RFLP and rhAmp	F:5′-TTGAATGAAGAGTTTCTGATGTTTT-3′ R: 5′CTCTGCATCAGCTGGACTGT-3′	*BsuRI*	[26]
*ABCB1* (NM_000927.3)	c.2677G > T	PCR-RFLP and TaqMan	F: 5′-TGCAGGCTATAGGTTCCAGG-3′ R: 5′-TTTAGTTTGACTCACCTTCCCG-3′	*BanI*	[27]
*PXR* (NM_022002.2)	c.69789A > G	PCR-RFLP and rhAmp	F: 5′-CACCATGCTTAGCTACAGCTCTATT-3′ R: 5′-GGCAAGATCACAACATGGGAAGA-3′	*BstDSI*	[28]
*PXR* (NM_022002.2)	c.63396C > T	PCR-RFLP	F: 5′-TGCTAGCAGTGCATAAGGGCTCAG-3′ R: 5′-TCCTGACCTTAGGTGATCCATGCC-3′	*Hpy188I*	[28]
*CYP2C19* (NM_000769.4)	c.681G > A	PCR-RFLP and rhAmp	F: 5′-AATTACAACCAGAGCTTGGC-3′ R: 5′-TATCACTTTCCATAAAAGCAAG-3′	*SmaI*	[29]
*CYP2C19* (NM_000769.4)	c.636G > A	PCR-RFLP	F: 5′-AAATTGTTTCCAATCATTTAGCT-3′ R: 5′-ACTTCAGGGCTTGGTCAATA-3′	*BamHI*	[30]

Cytochrome P450 oxidoreductase (*POR*), ATP Binding Cassette Subfamily B member 1 (*ABCB1*); Pregnane X Receptor (*PXR*).

**Table 2:** Genotype-phenotype relation of patient.

Gene	Variant	RefSNP (ID Number)	Patient Results		Refs.
			Genotype	Expected Phenotype	
CYP3A5 (NM_000777.5)	c.6986A > G	rs776746	GG	Non-expresser	[8]
CYP3A4 (NM_001202855.3)	g.-290A > G	rs2740574	AA	Wildtype. Normal activity	[10]
*CYP3A4* (NM_001202855.3)	c.15389G > T	rs35599367	CC	Wildtype. Normal activity	[11]
*CYP3A4* (NM_001202855.3)	c.1461_1462 insA	rs67666821	No insertion	Wildtype. Normal activity	[31]
*POR* (NM_000941.3)	c.1508C > T	rs1057868	CC	Wildtype. Normal activity	[32]
ABCB1 (NM_000927.3)	c.3435C > T	rs1045642	TT	TTT haplotype. P-gp decreased activity and expression.	[33]
*ABCB1* (NM_000927.3)	c.1236C > T	rs1128503	TT		
*ABCB1* (NM_000927.3)	c.2677G > T	rs2032582	TT		
*PXR* (NM_022002.2)	c.69789A > G	rs7643645	AG	Decreased activity of ABCB1 and CY3A	[34,35]
*PXR* (NM_022002.2)	c.63396C > T	rs2472677	CC	Wildtype. Normal activity	
*CYP2C19* (NM_000769.4)	c.681G > A	rs4244285	GG	Wildtype. Normal activity	[36]
*CYP2C19* (NM_000769.4)	c.636G > A	rs4986893	GG	Wildtype. Normal activity	

## Data Availability

No new data were created or analyzed in this study. Data sharing is not applicable to this article.
